# Supplementary material for: Into the spotlight: A spatial study of potentially underreported leptospirosis among dengue-negative patients in São Paulo city, Brazil
Source: PLoS Negl Trop Dis. 2025 Mar 5;19(3):e0012888. doi: 10.1371/journal.pntd.0012888 (PMC11922267; doi:10.1371/journal.pntd.0012888)
Supplement: S1 Table — The table summarizes the number (N) and percentage (%) of patients classified as positive or negative with spatial attributes including areas at risk of flooding, forest conservation units, slum areas, irregular settlements, slums undergoing urbanization, and urban versus rural locations. The results include p-values from chi-square tests to assess the association between patient status and spatial attributes, with confidence intervals (95% CI) provided for percentage estimates. (DOCX) [file pntd.0012888.s001.docx]

**Table S1: Distribution of Positive and Negative Patients by Spatial Attributes and Association Test Results.**

The table summarizes the number (N) and percentage (%) of patients classified as positive or negative with spatial attributes including areas at risk of flooding, forest conservation units, slum areas, irregular settlements, slums undergoing urbanization, and urban versus rural locations. The results include p-values from chi-square tests to assess the association between patient status and spatial attributes, with confidence intervals (95% CI) provided for percentage estimates.

|  | **Total** |  | **Positive** | | **Negative** | | **p-value** |
| --- | --- | --- | --- | --- | --- | --- | --- |
|  | **N** | **%** | **N** | **% (95%CI)** | **N** | **% (95%CI)** |  |
| **Areas at risk of flooding?** | | | | | | | |
| No | 5855 | 96.5 | 669 | 11.4 (10.6-12.26) | 5186 | 88.6 (87.7-89.37) | 0.813 |
| Yes | 211 | 3.5 | 23 | 10.9 (7.2-15.63) | 188 | 89.1 (84.4-92.77) |  |
| Total | 6066 |  | 692 | 11.4 (10.6-12.23) | 5374 | 88.6 (87.8-89.37) |  |
| **Forest conservation unit?** | | | | | | | |
| No | 5960 | 98.3 | 682 | 11.4 (10.7-12.27) | 5278 | 88.6 (87.7-89.35) | 0.519 |
| Yes | 106 | 1.7 | 10 | 9.4 (5-16.09) | 96 | 90.6 (83.9-95.05) |  |
| Total | 6066 |  | 692 | 11.4 (10.6-12.23) | 5374 | 88.6 (87.8-89.37) |  |
| **Located in slum areas?** | | | | | | | |
| No | 5594 | 92.2 | 640 | 11.4 (10.6-12.29) | 4954 | 88.6 (87.7-89.37) | 0.781 |
| Yes | 472 | 7.8 | 52 | 11 (8.4-14.08) | 420 | 89 (85.9-91.57) |  |
| Total | 6066 |  | 692 | 11.4 (10.6-12.23) | 5374 | 88.6 (87.8-89.37) |  |
| **Located in irregular settlements?** | | | | | | | |
| No | 4556 | 75.1 | 534 | 11.7 (10.8-12.68) | 4022 | 88.3 (87.3-89.19) | 0.183 |
| Yes | 1510 | 24.9 | 158 | 10.5 (9-12.08) | 1352 | 89.5 (87.9-91) |  |
| Total | 6066 |  | 692 | 11.4 (10.6-12.23) | 5374 | 88.6 (87.8-89.37) |  |
| **Located in slums urdergoing urbanization process?** | | | | | | | |
| No | 5983 | 98.6 | 684 | 11.4 (10.6-12.26) | 5299 | 88.6 (87.7-89.36) | 0.610 |
| Yes | 83 | 1.4 | 8 | 9.6 (4.7-17.36) | 75 | 90.4 (82.6-95.34) |  |
| Total | 6066 |  | 692 | 11.4 (10.6-12.23) | 5374 | 88.6 (87.8-89.37) |  |
| **Urban or rural area?** | | | | | | | |
| Rural Area | 31 | 0.5 | 4 | 12.9 (4.5-27.82) | 27 | 87.1 (72.2-95.48) | 0.788 |
| Urban Area | 5993 | 99.5 | 681 | 11.4 (10.6-12.19) | 5312 | 88.6 (87.8-89.42) |  |
| Total | 6024 |  | 685 | 11.4 (10.6-12.19) | 5339 | 88.6 (87.8-89.41) |  |
