# Supplementary material for: Into the spotlight: A spatial study of potentially underreported leptospirosis among dengue-negative patients in São Paulo city, Brazil
Source: PLoS Negl Trop Dis. 2025 Mar 5;19(3):e0012888. doi: 10.1371/journal.pntd.0012888 (PMC11922267; doi:10.1371/journal.pntd.0012888)
Supplement: Attachment — Submitted filename: Rebuttal_letter.docx [file pntd.0012888.s003.docx]

January 21^th^, 2025

Dear Editor,

We would like to express our gratitude the revision of our manuscript **“Into the spotlight: a spatial study of potentially underreported leptospirosis among dengue-negative patients in São Paulo city, Brazil”.** All formatting issues and technical items listed in the 'Journal Requirements' section have been addressed, including the addition of supplementary material containing the raw data from the study.

Please, find bellow our considerations according to each of the issues raised by the Reviewers.

**REVIEWER 1:**

**METHODS SECTION**

**Commentary #1:** The objective was clearly articulated and the study design was proper. The method section needs a minor revision to provide the details of the statistical test conducted. I have added few recommendation to improve the discussion section.

**RESPONSE:** We are very grateful for the reviewer’s comments, and hope that the revised manuscript has properly addressed this issue (page 18, Line 311 to 313).

**CONCLUSION SECTION**

**Commentary #1:** The conclusion needs a minor modification and authors are requested to address the comments included in the summary and general comments section.

**RESPONSE:** Based on the general comments provided, we hope that the conclusion of the revised version of the manuscript may address the reviewer’s suggestions.

**SUMMARY AND GENERAL COMMENTS**

**Commentary #1:** Esteves and the team conducted a spatial analysis to identify potentially underreported cases of leptospirosis among dengue-negative patients in São Paulo City, Brazil. The study analyzed 6,936 serum samples from patients who tested negative for dengue at public health services across São Paulo. These samples were subsequently tested for anti-leptospiral IgM serology to identify possible unreported leptospirosis cases. Results revealed that 786 (11.3%) samples were seroreactive for leptospirosis, with a higher prevalence observed in women than in men.
The spatial analysis identified 18 high-risk clusters for potentially undiagnosed leptospirosis, primarily located in peripheral areas and two prominent clusters in the northern and southern regions of the city. This investigation marks the first large-scale study highlighting potential hotspots and high-risk zones for unrecognized leptospirosis in São Paulo, a city that records approximately 4,000 reported cases annually.
While the study acknowledges certain limitations, the findings offer a critical foundation for designing and implementing targeted interventions to address the underdiagnosis of leptospirosis. This research holds significant public health implications and has the potential to draw the attention of Brazil's Ministry of Health. By addressing high-risk and underreported regions, such efforts could significantly contribute to the country's broader national health strategies, particularly as leptospirosis incidents rise globally, including in Brazil.

Few questions and recommendations are enlisted below:

1. This study successfully identified 11.3% of unrecognized leptospirosis cases from 6,937 dengue-negative serum samples collected from patients with febrile illnesses. What other potential illnesses could present the rest of the ~89% with symptoms similar to dengue or leptospirosis?

**RESPONSE:** This is indeed a very intriguing question. Most cases are likely associated with influenza, Zika, and COVID-19 viruses. However, we are currently conducting a study to investigate four other neglected diseases that are often overshadowed by dengue: leishmaniasis, Brazilian spotted fever, malaria, and Chagas disease. These potential etiologies are not discussed or mentioned in the present work to avoid unnecessary speculation about the possible occurrence of diseases with such significant public health implications.

2. Line 332: Please include the detais of statistical test that were conducted.

**RESPONSE:** The statistical tests conducted in the data are listed in page 18 lines 311 to 313 of the revised version.

1. What is the likelihood of detecting anti-leptospiral IgM positivity in serum from a patient exposed to saprophytic Leptospira, which are indigenous in nature, alongside pathogenic Leptospira? Authors are encouraged to reference studies (PMID: 39527098, 34249775, 35392072, 39018329) to emphasize the significance of their indigenous presence, evolutionary dynamics in nature, and their varied clinical outcomes that are similar to dengue or other diseases.

**RESPONSE:** It is certainly a difficult task to determine this likelihood. Yet, such interference is certainly possible. After careful reading of the suggested literature, a brief discussion on this issue was added. We hope that this issue has been properly addressed in the revised version (page 21 lines 505 to 512).

3. Line 324-328: What is the probability of identifying a seropositive cluster with saprophytic Leptospira infection, which, while not posing a direct threat to humans, could still induce seropositivity to anti-leptospiral IgM? Authors are requested to add that in the discussion around Line 467-471.

**RESPONSE:** Although this concept is undoubtedly interesting and relevant, our results do not allow us to explore this possibility any further. Addressing this issue would require distinguishing between true-positive and false-positive ELISA results. Such an analysis would be feasible if direct detection of leptospires had been performed, followed by genomic comparisons between saprophytic and pathogenic species. Direct detection in serum and clot samples is currently underway in samples taken from patients attended in 2024, as this project remains ongoing. We hope to publish these findings in the near future.

4. The authors are encouraged to discuss the presence of Leptospira in other clinical samples, such as genital specimens, to provide insights into additional transmission routes (e.g., PMID: 35446113). This discussion could aid future studies exploring the potential for sexual transmission and help determine whether there are any sex-based differences in leptospiral infection dynamics.

**RESPONSE:** We greatly appreciate the reviewer’s suggestion. While we find this inclusion intriguing, we believe it might be somewhat speculative and would contribute only minimally to the overall objectives of this work. This manuscript is primarily focused on highlighting that leptospirosis is a critical issue in São Paulo, one that is clearly being overlooked by local authorities. In future studies, a more targeted investigation into transmission pathways might be more appropriate, including insights into the venereal transmission.

5. Line 600-612: The authors have outlined the limitations of the study. It is recommended to include a section proposing more stringent diagnostic approaches to address the issues of misdiagnosis or underdiagnosis of leptospirosis. Incorporating advanced methods, such as molecular detection techniques or culture methods from serum samples, should be highlighted as potential strategies for more accurate and reliable confirmation of leptospirosis in future diagnostic protocols.

**RESPONSE:** We are very grateful for the reviewer’s comments. We hope that this issue has been properly addressed in the revised version (page 16 lines 642 to 650).

**REVIEWER 2:**

**METHODS SECTION**

**Commentary #1:** The ELISA method utilized in this study may be suitable for use in tertiary clinics and certain specific scenarios. However, for a cross-sectional study of this nature, the MAT would have been more appropriate. Given the number of samples available to the authors, not employing MAT is a missed opportunity, as it offers significantly more comprehensive information regarding the extent of infection, as well as the serovars or serogroups involved.

**RESPONSE:** We are very grateful for the reviewer’s comments. Indeed, the MAT was performed on samples from all patients who tested positive in the ELISA test. However, as the objective of the present study was not to compare these methods, these results were not included in the final version of the manuscript. Based on the suggestions and concerns raised by the reviewer, the MAT results have been included in this revised version. We hope that the inclusion of these results and discussion may address the reviewer’s concerns regarding MAT testing (Page 8, Lines 237 to 240; Page 11 Lines 364 to 372; Page 16, Lines 514 to 520).

The results of both tests revealed significant discrepancies, though. Among the 786 patients who tested positive in the ELISA test, only 7 were positive in the MAT. This divergence was anticipated, considering that nearly all patients in the study had serum samples tested between 6 and 10 days after symptom onset. Furthermore, the ELISA test is widely regarded as more suitable for detecting acute infections earlier than other serological techniques, including MAT, as highlighted in the introduction and discussion sections of the manuscript.

**SUMMARY AND GENERAL COMMENTS**

**Commentary #2:** This cross-sectional study by Bergmann Esteves and colleagues analyzed febrile, dengue-negative patients in São Paulo city for leptospiral antibodies using a commercially available ELISA. The results indicated that 11.3% of the tested patients were seropositive, and spatial analysis identified 18 high-risk clusters of potentially unrecognized leptospirosis in the city. A key concern is the choice of assay for the serosurvey. While the ELISA method used in this study may be appropriate for tertiary clinics and specific scenarios, the MAT would have been more suitable for a study of this nature. Given the substantial number of samples available to the authors, not employing MAT is a missed opportunity, as it is more specific and provides far more detailed information on the extent of infection and the specific serovars or serogroups involved.

**RESPONSE:** We appreciate the reviewer’s concern regarding the use of MAT. As previously mentioned, the MAT results have been included in the revised version of the manuscript (as also suggested by the Editor).

Although the MAT results were incorporated, it is important to emphasize that the primary objective of this study was to identify potentially underreported cases of leptospirosis among patients initially suspected of having dengue. Consequently, the study population primarily consisted of patients who were assessed between 6 and 10 days after the onset of nonspecific febrile symptoms. Given that this timeframe corresponds to the early stages of anti-Leptospira antibody formation in patients truly infected with leptospires, we prioritized the use of the technique with the highest potential for early infection detection (ELISA), performing MAT only on positive ELISA cases.

Therefore, we believe that the use of ELISA cannot be considered inappropriate. This choice was also based on the fact that patients with suspected dengue are typically not re-evaluated or retested following negative IgM dengue test results. As a result, there would be no opportunity to collect or analyze samples during the convalescent phase, imposing therefore the use of a test with superior performance for early detection in a single sample.

It is also important to emphasize that the study was not designed to compare the performance of the tests. Therefore, MAT testing was not conducted on patients who did not have IgM antibodies detected by the ELISA test. This comparison is currently underway, using samples collected from patients seen in 2024, and the results will be published in the future to ensure that the discussion regarding the performance of these tests is properly addressed.
